# Staying in Place: In Vitro Comparison of Extracorporeal Membrane Oxygenation Cannula Fixation for Dislodgment Prevention

**DOI:** 10.3390/jcm14051712

**Published:** 2025-03-04

**Authors:** Roxana Moayedifar, Johanna Schachl, Markus Königshofer, Martin Stoiber, Julia Riebandt, Daniel Zimpfer, Thomas Schlöglhofer

**Affiliations:** 1 Department for Cardiac and Thoracic Aortic Surgery, Medical University Vienna, 1090 Vienna, Austria; roxana.moayedifar@meduniwien.ac.at (R.M.); johanna.schachl@meduniwien.ac.at (J.S.); julia.riebandt@meduniwien.ac.at (J.R.); daniel.zimpfer@meduniwien.ac.at (D.Z.); 2 Center for Medical Physics and Biomedical Engineering, Medical University Vienna, 1090 Vienna, Austria; markus.koenigshofer@meduniwien.ac.at (M.K.); martin.stoiber@meduniwien.ac.at (M.S.); 3 Ludwig Boltzmann Institute for Cardiovascular Research, 1090 Vienna, Austria

**Keywords:** extracorporeal membrane oxygenation, cannula fixation, cannula dislodgment, securement, adhesive anchoring, cannula-related infection

## Abstract

**Background/Objectives**: Secure large-bore cannula insertion is critical for effective extracorporeal membrane oxygenation (ECMO), as inadequate fixation can lead to complications such as infection, dislodgment, and life-threatening events. With inconsistent guidelines for ECMO line management, this study compares the effectiveness of traditional suture fixation to an adhesive securement method in the prevention of ECMO cannula dislodgment using an in vitro model. **Methods**: Porcine skin and muscle tissue sections were prepared and mounted in a custom holder. A 21F venous ECMO cannula was inserted using a modified Seldinger technique. Three fixation methods were randomly compared: (1) three silk sutures, and (2a) one silk suture with a CathGrip adhesive anchoring device. In addition, a sub-analysis was performed using (2b) the Hollister adhesive anchoring device. A uniaxial testing machine simulated 50 mm cannula dislodgment, measuring tensile forces at 12.5, 25, and 50 mm dislodgment points. **Results**: A total of 26 ECMO cannula fixations using sutures, 26 with adhesive CathGrip, six with a Hollister device, and three controls were tested across six porcine samples. Sutures demonstrated greater variability in force at maximum dislocation, with 27% rupturing at 50 mm. In contrast, CathGrip provided greater flexibility without tearing. The adhesive exhibited higher stiffness (2.38 N/mm vs. 2.09 N/mm, *p* < 0.001) and dislodgment energy (0.034 J vs. 0.032 J, *p* = 0.002) in the 0–5 mm range, while sutures showed greater stiffness in the 5–50 mm range (1.42 N/mm vs. 1.18 N/mm, *p* < 0.001). At larger displacements (25 mm and 50 mm) and in total energy absorption, no statistically significant differences were observed (*p* = 0.57). In a sub-analysis, the six fixations using the Hollister device exhibited higher variability and significantly lower dislodgment forces at 25 mm (*p* = 0.033) and 50 mm (*p* = 0.004) compared to the CathGrip device. **Conclusions**: This study suggests that adhesive anchoring methods, such as CathGrip, may provide comparable or potentially superior fixation strength to sutures for ECMO cannula stabilization under controlled conditions. However, further research, including clinical trials, is necessary to confirm these findings, evaluate long-term performance, and explore the implications for dislodgment risk and infection prevention in clinical practice.

## 1. Introduction

Extracorporeal Membrane Oxygenation (ECMO) is a critical life-support therapy for patients with severe cardiac and respiratory failure [1,2,3]. This invasive technique requires the insertion of large-bore cannulas to facilitate extracorporeal blood oxygenation and circulation [2]. However, the success of peripheral ECMO therapy is highly dependent on the precise placement and secure fixation of these cannulas [2,4]. Inadequate cannula fixation can lead to significant complications, including impaired blood flow, infection, mechanical circuit dysfunction, and even cannula dislodgment, which may result in air embolism or catastrophic bleeding [2,4,5]. ECMO cannula infections occur more frequently than those in other vascular devices, with an incidence of 7.1 episodes per 1000 ECMO days [3]. Common pathogens include coagulase-negative staphylococci, Candida spp. Enterobacteriaceae, and Pseudomonas aeruginosa [6]. Prolonged ECMO duration and greater illness severity are key risk factors [7]. Additionally, cannula micro-motion at the entry site may further elevate infection risk [8]. In a 2019 global cross-sectional analysis, 34% of respondents reported cases of cannula malposition, dislodgment, or accidental decannulation at their centers within the previous five years, resulting in adverse patient outcomes. Among those incidents, more than half provided detailed accounts identifying inadequate cannula securement as the leading cause. Additionally, over three-quarters of survey participants indicated that the development of an international, evidence-based guideline for ECMO line management would be instrumental in enhancing bedside practices [2].

To prevent these complications, the clinical practice guidelines from the Extracorporeal Life Support Organization (ELSO) recommend securing ECMO cannulas to the skin at a minimum of two points, with regular monitoring of their positioning and stability [9]. However, the guidelines do not specify the optimal securement method. The most commonly used fixation technique involves securing the ECMO cannulas with sutures [2], typically utilizing horizontal mattress or purse-string sutures. In addition to sutures, some centers use securement devices, such as adhesive anchors, which grip the cannula and attach it to the skin [2,10]. Nonetheless, evidence on the effectiveness of these devices in ECMO cannula fixation remains limited [2,4]. A recent analysis of adhesive anchoring devices used for left ventricular assist device (LVAD) drivelines demonstrated a significant reduction in tensile forces at the driveline exit site, suggesting potential benefits for ECMO cannula fixation as well [11].

Despite the critical importance of secure ECMO cannula fixation, current guidelines and data remain inconsistent and lack standardization across clinical settings. However, only limited progress has been made in this area since then [2]. To address this gap, we conducted an in vitro study comparing the safety and efficacy of the traditional suture fixation method with alternative adhesive securement approaches for preventing ECMO cannula dislodgment. This study aims to provide a comprehensive understanding of the challenges and best practices in ECMO cannula fixation, with the goal of improving patient outcomes and reducing ECMO-related complications.

## 2. Materials and Methods

The setup was developed in accordance with the methods of Pearse et al. [4] and Bull et al. [3], who compared sutured ECMO cannulas to alternative fixation approaches, such as tissue adhesive.

**Figure 1:**
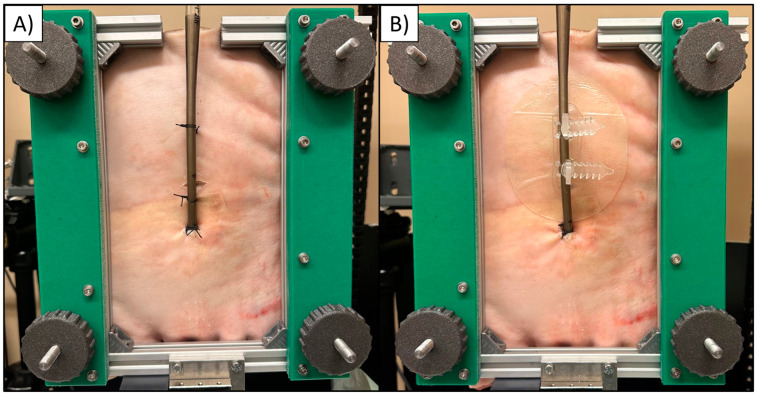
Techniques tested for ECMO cannula fixation: (**A**) standard (three sutures); (**B**) adhesive anchoring device (CathGrip) with one suture at the cannula insertion site.

**Figure 2:**
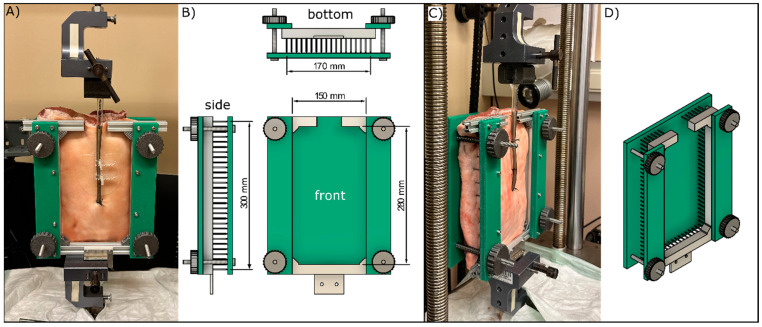
(**A**) Test specimen with implanted ECMO Cannula and adhesive fixation (front view); (**B**) front, side, and bottom views of the testing frame; (**C**) side view of a test specimen with implanted ECMO cannula and suture fixation; (**D**) side view of the testing frame.

To mimic the patients’ skin, six porcine skin and muscle tissue sections (20 × 30 × 4 cm) were obtained from adult pigs (processed at a slaughterhouse). After removing the hair from the skin and securing the tissue in a custom-made holder (Figure 1 and Figure 2), the skin was cleaned with an alcohol swab and warmed up with a heat gun for 1–2 min to approximately skin temperature, as the adhesion properties of the adhesive can be temperature-dependent [11,12]. Using a modified Seldinger technique, a 21F venous ECMO Cannula (Bio-Medicus Life Support, Medtronic Inc., Minneapolis, MN, USA) was inserted through the skin and into the test tissue up to the 45 cm cannula mark. The cannula was then secured (1:1 randomization) to the surrounding skin using one of the following three fixation methods:

(1) Three standard silk sutures (Sofsilk wax-coated braided silk, 30 inches, 75 cm, Coviden Ilc) each tied with 9–10 knots (see Supplemental Figure S1).
(2) (a) One silk suture at the cannula insertion (tied with 9–10 knots) combined with a CathGrip adhesive anchoring device (CathGrip Tube Securement Device, Hydrocolloid, Large, Double strap, Bravida Medical, Kaneville Ct, Geneva, IL, USA). (b) As a sub-analysis, we conducted six tests using one silk suture at the cannula insertion (tied with 9–10 knots) in combination with the Hollister adhesive anchoring device (Horizontal Tube Attachment Device, Hollister Incorporated, Libertyville, IL, USA; see Supplemental Figure S4)

After implanting the cannula, the tissue sample was mounted into a custom-made holder (Figure 2B,D), which was then secured in a uniaxial universal testing machine (Beta 10–2.5; Messphysik Materials Testing GmbH, Graz, Austria). The ECMO cannula was attached to a load cell (1 kN, Type TS, Class C2, AEP transducers, Cognento, Italy) and pulled upward to simulate a 50 mm extreme dislodgment at a speed of 250 mm/min. The load was measured at 12.5 (25% of maximal dislodgment), 25 (50% of maximal dislodgment), and 50 mm dislodgment for both fixation methods, along with reference measurements taken without any fixation. In addition, the stiffness, expressed in units of N/mm, was determined by calculating the slope of the load–displacement curve within two intervals: 0–5 mm (representing micro motions) and 5–50 mm (representing larger motions). Similarly, the dislodgment energy, represented by the area under the curve for these intervals, was calculated in joules (J).

A total of 26 fixations were performed using sutures, 26 using the adhesive CathGrip device, and six using the Hollister adhesive anchoring device (sub-analysis). Three reference measurements were obtained without any fixation, all conducted across six different porcine samples.

In addition, photographs taken before and after the measurement were analyzed using ImageJ 1.54d (Wayne Rasband and contributors, National Institutes of Health, Bethesda, MD, USA) to measure the distance between the cannula insertion site and the 45 cm mark, allowing for the determination of the actual displacement at maximal load.

### Statistical Analysis

Statistical analysis was performed using SPSS (IBM SPSS Statistics, Version 29.0, SPSS Inc., Chicago, IL, USA). Normally distributed variables are presented as mean ± standard deviation, while non-normally distributed data are reported as median (interquartile range [IQR]). The measured loads at 12.5 mm, 25 mm, and 50 mm dislodgment (F12.5, F25, and F50); the distance between cannula insertion site and the 45 cm cannula mark; the stiffness within the 0–5 mm and 5–50 mm ranges; and the dislodgment energy for these intervals, along with the total dislodgment energy, were tested for normality using the Shapiro–Wilk test. An unpaired t-test was used to compare normally distributed continuous variables, while the Mann–Whitney U test was applied for non-normally distributed data. Statistical significance was set at *p* < 0.05, with a Bonferroni correction applied to adjust for multiple comparisons (*p* < 0.017).

## 3. Results

### 3.1. Dislocation Force and Flexibility: Sutures and CathGrip

**Figure 3:**
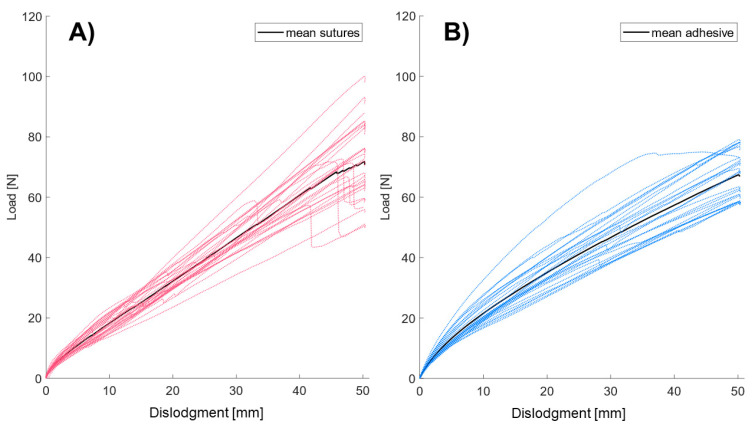
Individual and mean load per dislodgment distance for sutures (**A**) and the CathGrip adhesive anchoring device (**B**).

**Figure 4:**
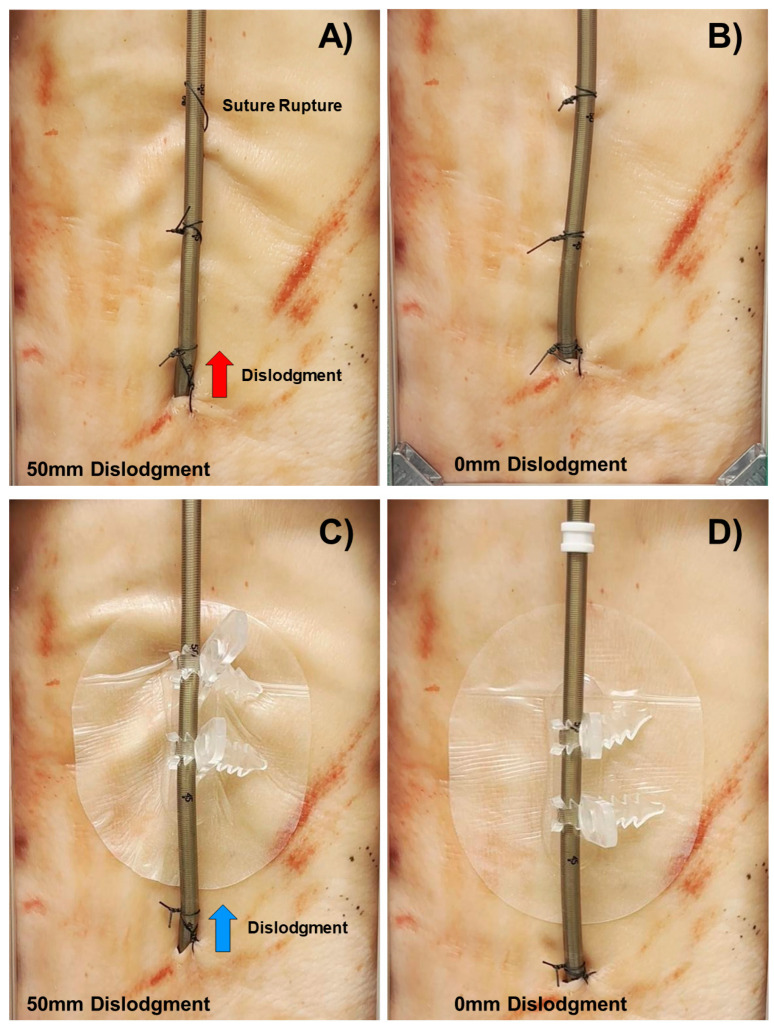
Depiction of standardized suture fixation with three sutures at 50 mm dislodgment of the ECMO cannula (**A**); at 0 mm dislodgment (**B**); and after adhesive anchoring device (CathGrip) fixation at 50 mm ECMO cannula dislodgment (**C**) as well as 0 mm dislodgment (**D**).

Sutures demonstrated greater variability in the force required for maximum dislocation (at 50 mm), despite consistent application by a single surgeon (Figure 3A,B). In addition, sutures exhibited higher stiffness, with 27% rupturing at 50 mm of displacement (see Figure 4A) and often tearing at the point of maximum force (Fmax). In contrast, CathGrip adhesive anchoring showed greater flexibility and maintained structural integrity under similar conditions (Figure 4C,D), suggesting a more resilient and adaptable option for securing ECMO cannulas compared to sutures.

The results demonstrate significant differences between sutures and adhesive in terms of stiffness and dislodgment energy (Table 1). In the 0–5 mm range, the CathGrip adhesive exhibits a higher stiffness (2.38 N/mm vs. 2.09 N/mm, *p* < 0.001) and higher dislodgment energy (0.034 J vs. 0.032 J, *p* = 0.002), indicating greater resistance to deformation in this initial range. However, within the 5–50 mm range, sutures exhibited a higher stiffness (1.42 N/mm vs. 1.18 N/mm, *p* < 0.001), indicating enhanced performance in resisting deformation at larger displacements. Total energy absorption shows no statistically significant difference between the two methods (*p* = 0.57), indicating comparable overall energy storage capabilities.

### 3.2. Cannula Displacement

**Figure 5:**
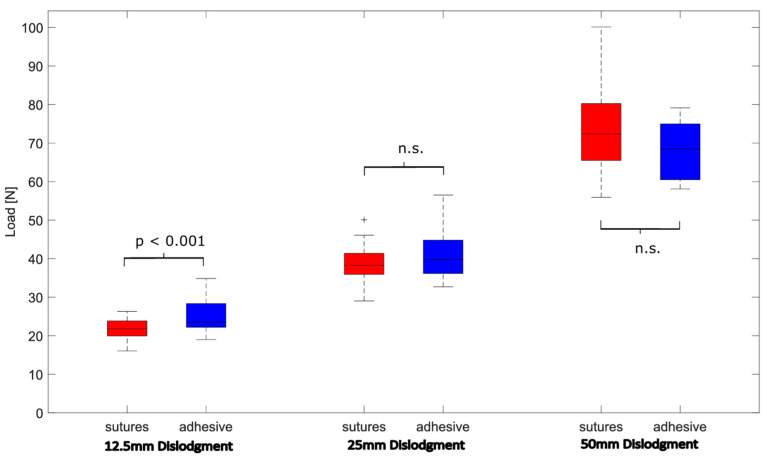
Statistical comparison of extraction load at 12.5 mm, 25 mm, and 50 mm cannula dislodgment, stratified by sutures (red) and the adhesive anchoring device (blue). n.s. (not statistically significant).

When comparing ECMO cannula fixation using an adhesive anchoring device to the traditional method of three skin sutures, the force required to displace the cannula by 12.5 mm was significantly higher with the adhesive device vs. sutures (24.8 ± 4.1 N vs. 21.2 ± 2.8 N, *p* < 0.001). However, at 25 mm (40.9 ± 6.0 N vs. 38.7 ± 4.5 N, *p* = 0.15) and 50 mm (68.4 (15.3) N vs. 72.4 (18.3) N, *p* = 0.04) of cannula dislodgment, both fixation methods demonstrated comparable performance, with no significant differences in extraction force (Figure 5).

In the absence of sutures or an adhesive anchoring device, the force required to displace the cannula was significantly lower (*p* < 0.001) compared to all of the fixation methods (12.5 mm: 3.9 ± 0.02 N, 25 mm: 3.7 ± 0.02 N, 50 mm: 3.2 ± 0.2 N), as detailed in Supplementary Material, Figure S3.

No significant differences were observed between the actual cannula displacement distance (Figure 4A,C) under maximal load for adhesive anchoring and sutures (13.5 ± 3.9 mm vs. 13.6 ± 4.2 mm, *p* = 0.97).

### 3.3. Sub-Analysis: Comparison of CathGrip and Hollister Performance

In a supplementary analysis, the CathGrip adhesive anchoring device was compared with the Hollister fastening device (see Supplementary Figure S4), an alternative adhesive device for ECMO cannula fixation. The force required to dislodge the cannula at 12.5 mm was not significantly different (median 23.5 N [IQR 6.2] vs. 21.5 N [IQR 5.4], *p* = 0.324). However, at 25 mm displacement, CathGrip required a significantly higher force (median 39.7 N [IQR 9.0] vs. 33.9 N [IQR 4.3], *p* = 0.033), and at 50 mm, the difference was even more pronounced (median 68.4 N [IQR 15.3] vs. 55.6 N [IQR 14.8], *p* = 0.004).

## 4. Discussion

The secure fixation of ECMO cannulas is crucial for preventing serious complications such as dislodgment, bleeding, and circuit malfunction [2,5]. Despite its importance, there is a lack of comprehensive data on the optimal fixation techniques for these cannulas [2,4].

Our study highlights distinct differences between traditional suturing and adhesive anchoring for ECMO cannula fixation. Sutures, although widely used, exhibited notable variability in performance, displaying a more inhomogeneous load/dislodgment curve, higher stiffness in the 5–50 mm range (1.42 N/mm vs. 1.18 N/mm, *p* < 0.001), and a 27% rupture rate at 50 mm. In contrast, the adhesive anchoring device (CathGrip) provided superior flexibility and required significantly more force to achieve 12.5 mm displacement, suggesting enhanced stability against small displacements. Beyond 12.5 mm, both methods performed similarly, as total energy absorption was comparable (*p* = 0.57), indicating similar overall energy storage capabilities.

Maintaining cannula stability throughout ECMO support is a critical patient safety priority, as decannulation can lead to life-threatening emergencies [2,3]. Previous studies have identified factors such as ambulation, transport, and routine patient care as contributing to cannula dislodgment, often due to suboptimal dressing and securement techniques [2]. The current shift towards reduced sedation and early mobilization in specific patient groups further emphasizes the need for secure cannula fixation, placing additional demands on the methods used [2,13]. Moreover, suture fixation can lead to skin tearing or irritation, particularly in patients with fragile skin, resulting in pain and discomfort while also increasing the risk of infection at the insertion site [2]. This underscores the need to balance patient safety with comfort in ECMO cannula securement. Adhesive anchoring devices present a promising alternative, offering potential benefits for both stability and patient comfort [2]. Although various centers employ different adhesive devices for cannula stabilization, many commercial products, such as “Grip-Lok” style devices, Foley catheter devices, and colostomy ring patches, are not specifically designed for ECMO use, emphasizing the need for better-suited solutions and rigorous testing of available products [2,14].

One of the securement devices used in this study, CathGrip, is designed for tubing sizes 6–42 Fr and utilizes a latex-free hydrocolloid material compatible with various skin types. It provides flexibility and stability, with a wear time of up to 7 days, making it a practical option for prolonged use. A recent analysis of adhesive anchoring systems for LVAD driveline stabilization found that CathGrip not only exhibited the lowest tensile forces at the driveline exit site but also provided the highest level of protection compared to seven alternative products [11]. This immobilization technique effectively prevents trauma to the exit site, potentially reducing the risk of driveline infections, a serious complication in LVAD therapy [11]. Conventional sutures, which require multiple wraps around the cannula before being tightened, introduce the risk of potentially damaging constriction to the cannula. In contrast, a key advantage of the CathGrip is its double soft straps with a no-slip grip, providing secure fixation without the risk of constriction or harm to the tubing. Notably, the Hollister, a widely used adhesive anchoring device, demonstrated greater variability, with frequent detachment events that may undermine its reliability as a secure fixation method for ECMO cannulas in clinical practice (see Supplementary Figure S4). In contrast, CathGrip required significantly higher (=better) dislodgment force at 25 and 50 mm compared to Hollister.

Another key finding of this study is that the force required to dislodge the ECMO cannula by 12.5 mm was significantly greater (=better) with the CathGrip adhesive securement compared to sutures. This suggests that smaller cannula movements may be more effectively prevented with adhesive fixation rather than sutures. Small ECMO cannula movements, or “micro-motions,” were first described by Luchini et al. in 2021 as contributors to local infection, with their management protocol limiting these movements to 5 mm [8,15]. However, this was based on a small study focused primarily on prone positioning, and such micro-motions are likely to exceed 5 mm during routine activities like bathing, ambulation, and patient transport. The data from our study demonstrate that, particularly in the highly relevant micro-motion range of up to 5 mm, the CathGrip adhesive anchoring exhibits significantly greater stiffness (2.38 N/mm vs. 2.09 N/mm, *p* < 0.001) and higher dislodgment energy (0.034 J vs. 0.032 J, *p* = 0.002), indicating superior resistance to deformation (Table 1). These findings suggest that the CathGrip adhesive provides enhanced mechanical stability under small-scale displacements, a critical factor in reducing the risk of catheter dislodgment during clinical use. Another recent study explored the impact of small dual-lumen cannula movements in veno-venous ECMO patients, showing that while minor displacements do not significantly increase recirculation as long as the return jet remains in the right atrium, they do elevate the risk of thrombosis due to increased wall shear stress and turbulent kinetic energy [15]. Similarly, a 2018 in vitro study demonstrated that tissue adhesive not only effectively prevented bacterial migration to the ECMO cannula insertion site but also significantly increased the force required to dislodge the cannula. When used alone or combined with a transparent dressing, adhesives were successful in preventing bacterial migration along the cannula tunnel [3]. These findings underscore the critical need for secure, effective cannula fixation methods to minimize the risks of both infection and thrombosis during ECMO therapy.

A recent in vitro analysis of cyanoacrylate tissue adhesives was the first to demonstrate that securement using sutures and n-butyl-2-octyl cyanoacrylate significantly increased the force required to dislodge an ECMO cannula compared to transparent polyurethane dressings and 2-octyl cyanoacrylate [4]. Sutures also provided greater flexibility compared to cyanoacrylate adhesives. Both cyanoacrylate formulations preserved the cannula’s resistance strength after 60 min, indicating that n-butyl-2-octyl cyanoacrylate is a strong option for adjunct ECMO cannula fixation [4]. When comparing our study on CathGrip with previous research, notable differences emerge. Both securement methods effectively stabilized the cannula; however, CathGrip offered superior flexibility compared to sutures, addressing the rigidity observed with cyanoacrylate adhesives. This enhanced flexibility of CathGrip is particularly beneficial in scenarios involving patient movement, as it minimizes the risk of skin irritation or tearing—a factor not examined in the cyanoacrylate study. Additionally, CathGrip required a significantly higher dislodgment force compared to sutures at 12.5 mm displacement, consistent with the strength of n-butyl-2-octyl cyanoacrylate. Importantly, neither study reported any compromise to cannula integrity with either securement method. Furthermore, CathGrip’s use of gentle hydrocolloid materials, designed to enhance patient comfort, offers a distinct clinical advantage, particularly for patients with sensitive skin or those undergoing early mobilization. These benefits may extend beyond ECMO cannulas to other exit site securements, such as chest tubes, percutaneous endoscopic gastrotomy (PEG) tubes, and central venous catheters. As such, CathGrip presents a compelling solution for optimizing securement strategies across a range of medical devices by combining robust fixation, flexibility, and enhanced patient comfort.

In summary, our findings suggest that CathGrip adhesive anchoring provides clinical benefits such as superior flexibility and stiffness, as well as resistance to micro-motions, reducing the risks of skin tearing, cannula constriction, and infection, which can improve patient comfort and facilitate early mobilization. This is supported by our results, which show the following: (1) Adhesive anchoring required significantly higher force to achieve 12.5 mm displacement (24.8 ± 4.1 N vs. 21.2 ± 2.8 N, *p* < 0.001). (2) In the 0–5 mm range, CathGrip showed higher stiffness (2.38 vs. 2.09 N/mm, *p* < 0.001) and dislodgment energy (0.034 vs. 0.032 J, *p* = 0.002), indicating better resistance to micro-motions. (3) In the 5–50 mm range, sutures were stiffer (1.42 vs. 1.18 N/mm, *p* < 0.001) but had high variability with a 27% rupture rate, while overall energy absorption was comparable (*p* = 0.57).

## 5. Conclusions

Adhesive anchoring represents a promising alternative for securing ECMO cannula insertion sites. The CathGrip adhesive anchor may provide fixation strength comparable to or even superior to that of standard methods, such as skin sutures, potentially reducing the risks associated with dislodgment. However, future clinical research should focus on correlating in vivo ECMO cannula dislodgment and infection rates with various fixation methods. A randomized clinical trial would yield valuable evidence regarding the effectiveness and ease of use of the adhesive anchoring device for ECMO cannula securement.

## 6. Limitations

This study has several limitations. First, it utilized a porcine skin model, which may not fully reflect the complexities of human skin, as human skin can vary significantly due to factors such as age, health, skincare routine, and environmental exposure. Second, the model does not account for variations in skin properties, such as aging or hydration levels, which can affect adhesion. Additionally, skin temperature was only approximated, potentially influencing adhesive performance. Third, the depth of the stitches was not standardized or controlled, which could influence the fixation strength and may not perfectly replicate clinical practice. Fourth, the quality of the porcine tissue, such as variability in thickness and elasticity, could have impacted the results and may not fully represent in vivo conditions. Fifth, while the overall energy storage capabilities were similar, this metric alone does not capture other clinically relevant factors, such as the ability to resist micro-displacements or maintain stability under specific forces. Future studies could explore whether this equivalence holds under more diverse mechanical conditions, such as varying load angles or prolonged stress exposure. Sixth, this study was limited to short-term testing, which may not reflect differences that could emerge over prolonged usage, such as degradation of adhesive performance or material fatigue. While the securement device used in this study is designed for a wear time of up to seven days, further investigation into its long-term performance under realistic clinical conditions is needed, including a detailed cost-effectiveness analysis. Lastly, the small sample size limits the generalizability of the findings, necessitating further studies with larger, more diverse samples to validate these results.

## Figures and Tables

**Table 1:** Stiffness and dislodgment energy with sutures and the adhesive anchoring device for micro- and larger cannula dislodgment. SD, standard deviation; IQR, interquartile range; J, joule.

Variable Mean ± SD or Median [IQR]	Sutures	Adhesive	*p*-Value
Stiffness _0–5mm_ (N/mm)	2.09 [0.58]	2.38 [0.82]	<0.001
Stiffness _5–50mm_ (N/mm)	1.42 ± 0.24	1.18 ± 0.15	<0.001
Energy _0–5mm_ (J)	0.032 [0.01]	0.034 [0.01]	0.002
Energy _5–50mm_ (J)	1.89 ± 0.25	1.93 ± 0.24	0.62
Energy _Total_ (J)	1.92 ± 0.25	1.96 ± 0.25	0.57

## Data Availability

The data presented in this study are available on request from the corresponding author. The data are not publicly available due to privacy restrictions.

## Supplementary Materials

The following supporting information can be downloaded at: https://www.mdpi.com/article/10.3390/jcm14051712/s1. Figure S1: Detailed close-up image showing the tied 9–10 knots used in the suture group. Figure S2: Example data curve, in which the slope of the curve is drawn in the 0–5 mm and 5–50 mm ranges, corresponding to the respective stiffness. The areas under the curve in these ranges correspond to the respective dislodgment energy. Figure S3: Mean load vs. dislodgment and 95% confidence intervals for sutures (red), CathGrip (blue), and Hollister (yellow) adhesive anchoring devices, with no fixation as a reference (green). Figure S4: Depiction of adhesive anchoring device (Hollister) fixation at 0 mm ECMO cannula dislodgment (A) and 50 mm dislodgment (B), along with a statistical comparison (C) of extraction load at 12.5 mm, 25 mm, and 50 mm cannula dislodgment, stratified by CathGrip (blue) and Hollister (yellow) adhesive anchoring devices.
