# Supplementary material for: Staying in Place: In Vitro Comparison of Extracorporeal Membrane Oxygenation Cannula Fixation for Dislodgment Prevention
Source: J Clin Med. 2025 Mar 4;14(5):1712. doi: 10.3390/jcm14051712 (PMC11901029; doi:10.3390/jcm14051712)
Supplement: Supplementary file 1 [file jcm-14-01712-s001.zip › jcm-3442442-supplementary.pdf]

Journal of
Clinical Medicine

MDPI

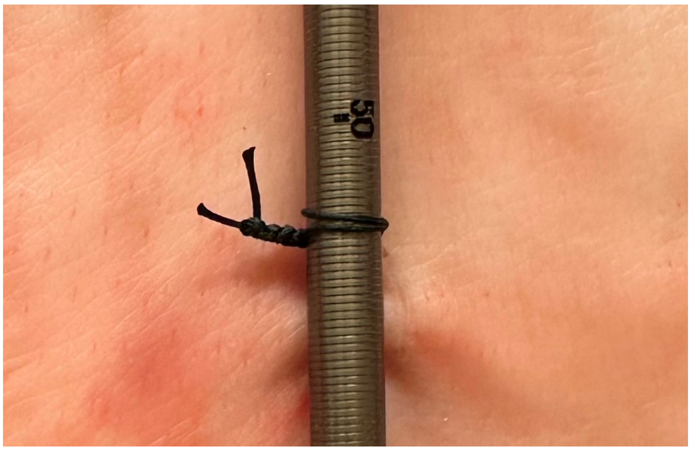

Figure S1. Detailed close-up image showing the tied 9-10 knots used in the suture group.

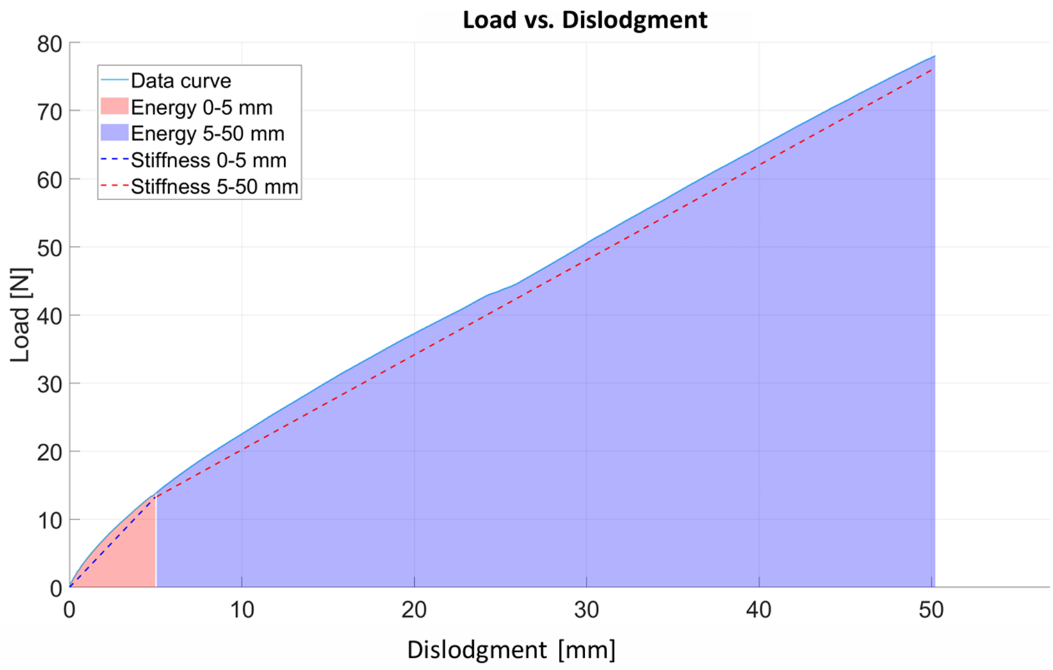

Figure S2. Example data curve, in this is drawn the slope of the curve in the range 0-5mm and 5-50mm, which correspond to the respective stiffness and the areas under the curve in the range 0-5mm and 5-50mm which correspond to the respective dislodgment energy.

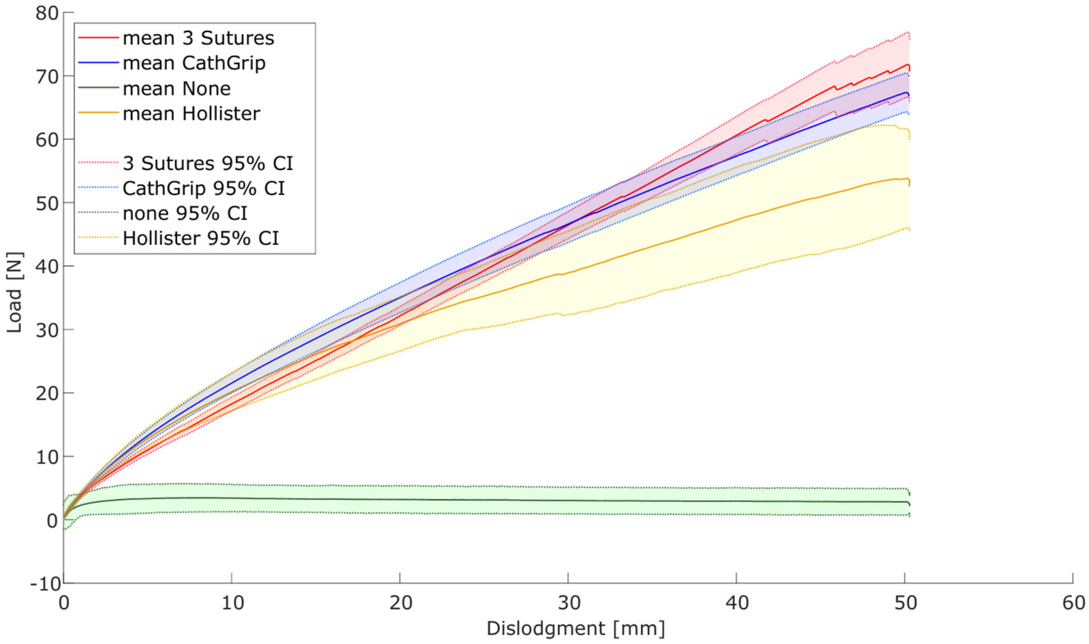

Figure S3. Mean load vs. dislodgment and 95% confidence intervals for sutures (red), CathGrip (blue) and Hollister (yellow) adhesive anchoring devices, and no fixation as reference (green).

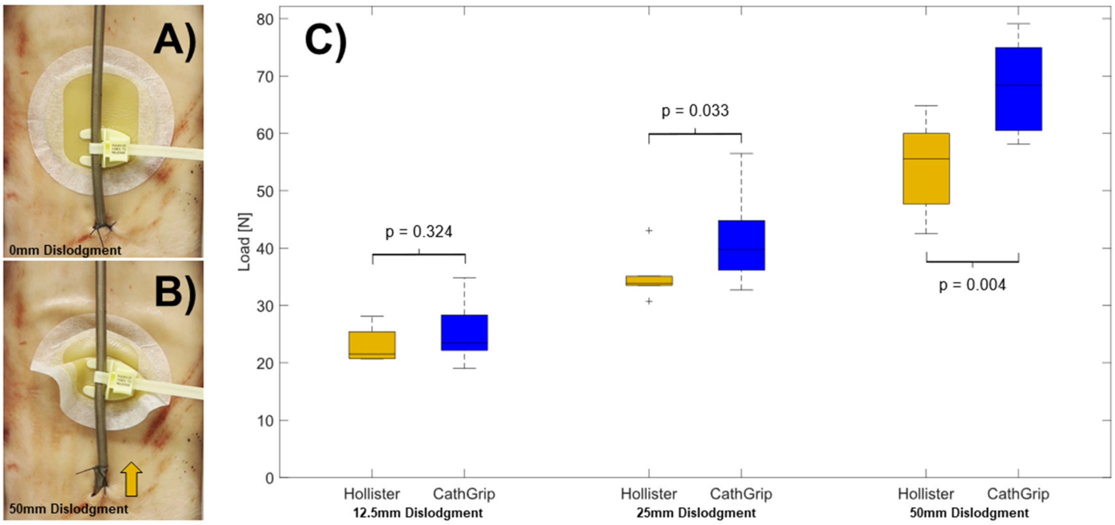

Figure S4. Depiction of adhesive anchoring device (Hollister) fixation at 0mm ECMO cannula dislodgment (A) as well as 50mm dislodgment (B), and statistical comparison (C) of extraction load at 12.5 mm, 25 mm, and 50 mm cannula dislodgment, stratified by CathGrip (blue) and Hollister (yellow) adhesive anchoring devices.
